# Impact of the COVID-19 pandemic on career intention amongst undergraduate medical students: a single-centre cross-sectional study conducted in Hubei Province

**DOI:** 10.1186/s12909-022-03201-4

**Published:** 2022-03-08

**Authors:** Xue-lin Wang, Ming-xiu Liu, Shuai Peng, Lei Yang, Chen Lu, Shi-cong Shou, Jian-ru Wang, Jun-yi Sun, Jia-qi Wang, Yan Hu, Jun Zhao, Peng Duan

**Affiliations:** 1 Department of Obstetrics and Gynaecology, Xiangyang No.1 People’s Hospital, Hubei University of Medicine, Xiangyang, 441000 China; 2 School of Nursing, Hubei University of Medicine, Shiyan, 442000 China; 3 School of Fourth Clinical, Hubei University of Medicine, XiangYang, 441000 China; 4 School of Public Health, Hubei University of Medicine, Shiyan, 442000 China

**Keywords:** Medical education, Undergraduate students, Career intention, COVID-19, Cross-sectional study

## Abstract

**Background:**

Undergraduate medical (UM) students faced the difficulties inherent in medical careers due to the coronavirus (COVID-19) outbreak. Thus, imperative containment measures might affect UM students’ career intentions. Information on the factors that may be associated with these students’ career change intentions is limited.

**Methods:**

We conducted a cross-sectional survey in August 2020 to investigate the impact of the COVID-19 pandemic on career intention and the associated factors in UM students. Univariate analyses and logistic regression analysis were performed to identify said factors.

**Results:**

A total of 2040 medical students from the Hubei University of Medicine were surveyed. Univariate analyses showed that grade, attitude towards healthcare, and the degree of the COVID-19 pandemic’s impact on the students’ lives were associated with changes in career choice (*P*<0.05). Logistic regression analysis showed that Grade 2, Grade 5, attitude towards a medical career, and having relatives with a medical background were associated with changes in career choice. The degree of the COVID-19 pandemic’s impact was a common and significant factor associated with career preference, career perspective, and ideal workplace.

**Conclusions:**

Changes in career intentions were particularly influenced by grade, attitude towards being a health worker, and the degree of COVID-19’s impact on the participants’ lives. Treating large-scale public health emergencies rationally, setting up correct views of occupation choice, and building reasonable career planning may reduce the loss of medical talent.

**Supplementary Information:**

The online version contains supplementary material available at 10.1186/s12909-022-03201-4.

## Introduction

The intention or desire to engage in a certain career comes from an inner motivation [1]. Making a particular career choice boosts an individuals’ passion for work and leads them to achieve greater success [2]. Therefore, undergraduate medical (UM) students’ longing for and identification with the profession and their willingness to be healthcare practitioners will have an impact on their future career choice and medical practice. This identification may, in turn, affect the number of doctors in society and their degree of professionalism. A retrospective study showed that from the beginning of 2005 to the end of 2014, China produced 4,314,791 five-year clinical medical graduates and 413,186 five-plus-two programme medical graduates: a total of 4,727,977 clinical medical graduates. However, during this period, there was an increase of only 752,233 (15.91%) in the total number of registered clinical physicians in practice [3]. The study confirms a high attrition rate amongst medical graduates and physicians in China over the past 10 years. In China, the doctor-patient relationship still does not engender optimism [4]. Factors such as individual cognition, doctor-patient conflict, perceptions of the medical practice environment, salary level, and expectations regarding doctor-patient relationships influence whether medical students decide to pursue a career in the profession [4, 5].

At the end of 2019, the outbreak of a new coronavirus, which was named COVID-19, aroused global attention. The World Health Organization (WHO) characterized the COVID-19 outbreak as a pandemic [6]. The global pandemic continued to spread in 2021. Worldometers [7] data show that the global accumulated number of definite cases reached more than 160 million and the death toll reached more than 3 million by May 13, 2021. As a result, people worldwide have been under enormous pressure and many have experienced severe psychological distress [8, 9]. A series of studies on the effects of COVID-19 on physical health have emerged. The significance of disastrous events on a community can help evaluate citizens’ value systems. After the Wenchuan Earthquake in China, social values in the disaster-stricken areas dominated university students’ expressions of their value systems [10]. The continued impact of the pandemic had a knock-on effect on every aspect of college students’ lives [11, 12], especially per the psychological impact on their professional value intentions and life choices [12, 13].

We hypothesized in the present study that COVID-19 may have influenced medical students’ intentions to practice medicine. For this purpose, a cross-sectional online survey was conducted amongst UM students from the Hubei University of Medicine. We examined the effect of the pandemic on UM students’ career intentions and the relevant factors that influenced those intentions.

## Methods

### Study design and population

The present study was cross-sectional. The data were gathered through an online questionnaire that was developed, reviewed, and piloted by Dr. Peng Duan and his colleagues. We recruited 30 18-25-year-old UM students (70% of whom were female) from Grades 1 to 5 at Hubei University of Medicine to assess the feasibility of the questionnaire. Each participant was informed of the purpose of the research (i.e., to investigate the impact of COVID-19 on the participants’ career intentions). They were asked to give their consent to the collection of the data when they completed and submitted the anonymous and self-administered questionnaire.

The questionnaire was distributed through the Chinese professional survey website *Wenjuanxing* (http://www.wjx.com, Changsha Ranxing Information Technology Co., LTD, Changsha, China). The survey was completed online, and a number (only use to knock out duplicate respondents) was automatically provided by the website after they submitted their responses. The questionnaires were distributed between August 14 and August 28, 2020, through the widely used WeChat platform. We calculated the sample size using a sample rate of 5%, an allowable error of 1%, and a 5% significance level. The number of participants required was 1825. Clinical grades range from 1 to 5 and nursing grades from 1 to 4. All students were required to have practised in the hospital during the previous school year. The study protocol was approved by the Ethics Committee Board at the Hubei University of Medicine (No.2020-TH-062).

### Variables and measurement

The online survey comprised three main sections: (1) socio-demographic information; (2) degree of concern about the COVID-19 pandemic; and (3) whether career intention changed during the COVID-19 pandemic. Socio-demographic characteristics comprised age; gender; parents’ level of education; place of residence; monthly household income; monthly personal expenses; grade; specialty; relatives’ medical background; and parents’ attitude toward medical studies. The degree of concern about COVID-19 was estimated through the time spent focusing on the pandemic and subjective feelings and their impact on daily life. The participants were asked to assess the overall effect of the COVID-19 pandemic on their daily lives. Items included learning environment; sleep quality; diet; ability to travel; happiness; daily routine; and psychology. One point was recorded when each item was selected. A total score (0-7, Supplementary Table 1) was then calculated by summing the points. The sub-section on change of career intention comprised career choice; career preference; career perspective; and ideal workplace.

### Data collection and quality control

The questionnaire was administered over 2 weeks. First, the researchers contacted the UM students in each class. They explained the procedure and sent the students a link to the electronic questionnaire via WeChat. Each participant completed the survey anonymously. An Excel file was generated after the entire sample had submitted their responses. Upon analysis, we combined all Excel files and saved them as an Excel workbook file.

To avoid repetition, participants who filled out questionnaires with the same number (*n* = 27) were excluded from further analysis. Questionnaires indicating that the respondents were older than 30 or younger than 17 were regarded as invalid (*n* = 7). Four postgraduate students were also excluded. After these exclusions, a total of 2040 participants remained, yielding an effective response rate of 98.4%.

### Data analysis

Data analysis was performed by using R software version 4.0.2 (R Core Team [2020]. R: A language and environment for statistical computing. R Foundation for Statistical Computing, Vienna, Austria. URL https://www.R-project.org/). The Shapiro-Wilk test was used to examine if the variables were normally distributed. Descriptive statistics were used to represent the demographics of the participants and their concerns regarding COVID-19. We made some adjustments to the variables to improve the survey. The level of education of the parents was divided into four groups: middle school and below; high school; college; and university and above. We evaluated the degree of the pandemic’s impact on each UM student, with higher scores indicating a greater impact.

Continuous variables were reported as a mean, and standard deviation and categorical data were proportions. The difference between the categorical variables was assessed by using the chi-square test, while the student’s *t*-test was used to evaluate the variance in continuous variables. The significance level was set at 0.05, and two-tailed *p*-values were reported.

Logistic regression analysis was performed by using multiple covariates as independent variables and career intention change during the pandemic as an outcome variable. Multiple covariates and potential confounders were controlled to measure the association of change of career intention during COVID-19. These covariates included age; sex; residency; parents’ level of education; monthly family income; monthly personal expenses; grade; major; time spent concerned about COVID-19; viewing healthcare work as dangerous; having relatives with a medical background; parents’ attitude towards medical schools; and the degree of COVID-19’s impact. Odds ratios and 95% confidence intervals were reported. Stratified analysis was conducted by categorizing the participants according to specialty (i.e., clinical medicine or nursing). The association between specialty and changes in career intention was displayed using a forest plot.

## Results

### Demographics

A total of 2040 participants (Males = 546, Females = 1494) completed the survey. The mean age of participants was 20.4 ± 1.4 years. Most participants (1386, 67.9%) were Hubei residents, and 55.6% were from rural areas. They were divided into three groups: clinical medicine (803, 39.4%), nursing (989, 48.5%), and others (248, 12.2%). The score for the impact degree of the pandemic was 3.5 ± 1.6. The participants’ complete demographic details are shown in Table 1.

**Table 1:** Socio-demographic characteristics of medical students and univariate analysis of changes in career choice during the COVID-19 pandemic (*n* = 2040)

Variable	Total (*n* = 2040)	Change of career choice during COVID-19^a^		*P* value
		No (*n* = 1796)	Yes (*n* = 147)	
Age (year)				
mean (SD)	20.4 (1.4)	20.4 (1.4)	20.6 (1.4)	0.112
Sex				
Male	546 (26.8)	478 (26.6)	44 (29.9)	0.438
Female	1494 (73.2)	1318 (73.4)	103 (70.1)	
Residency				
Hubei rural	763 (37.4)	667 (37.1)	61 (41.5)	
Hubei urban	623 (30.5)	540 (30.1)	45 (30.6)	0.375
Rural other than Hubei	371 (18.2)	328 (18.3)	27 (18.4)	
Urban other than Hubei	283 (13.9)	261 (14.5)	14 (9.5)	
Educational level of parents				
Middle school or below	958 (47.0)	848 (47.2)	68 (46.3)	
High school	577 (28.3)	501 (27.9)	43 (29.3)	0.939
College	190 (9.3)	169 (9.4)	12 (8.2)	
University or above	315 (15.4)	278 (15.5)	24 (16.3)	
Family monthly income (CYN)				
< ¥3000	394 (19.3)	344 (19.2)	32 (21.8)	
¥3000-¥5999	819 (40.1)	718 (40.0)	62 (42.2)	0.683
¥6000-11,999	626 (30.7)	555 (30.9)	41 (27.9)	
≥¥12,000	201 (9.9)	179 (10.0)	12 (8.2)	
Monthly expense (CYN)				
< ¥500	66 (3.2)	53 (3.0)	8 (5.4)	
¥500-¥999	759 (37.2)	670 (37.3)	52 (35.4)	0.412
¥1000-¥1499	918 (45.0)	812 (45.2)	65 (44.2)	
≥¥1500	297 (14.6)	261 (14.5)	22 (15.0)	
Grade				
Grade 1	435 (21.3)	398 (22.2)	20 (13.6)	**0.008**
Grade 2	673 (33)	572 (31.8)	61 (41.5)	
Grade 3	484 (23.7)	436 (24.3)	30 (20.4)	
Grade 4	306 (15)	267 (14.9)	19 (12.9)	
Grade 5	142 (7.0)	123 (6.8)	17 (11.6)	
Major				
Clinical medicine	803 (39.4)	729 (40.6)	58 (39.5)	0.089
Nursing	989 (48.5)	845 (47.0)	79 (53.7)	
Others	248 (12.2)	222 (12.4)	10 (6.8)	
Time of concern about COVID-19 (minutes)				
< 5	341 (16.7)	290 (16.1)	31 (21.1)	
5-9	533 (26.1)	479 (26.7)	28 (19.0)	0.082
10-29	761 (37.3)	672 (37.4)	52 (35.4)	
≥30	405 (19.9)	355 (19.8)	36 (24.5)	
Regard healthcare as dangerous job				
No	881 (43.2)	797 (44.4)	50 (34.0)	**0.019**
Yes	1159 (56.8)	999 (55.6)	97 (66.0)	
Have relatives with medical background				
No	1594 (78.1)	1409 (78.5)	107 (72.8)	0.136
Yes	446 (21.9)	387 (21.5)	40 (27.2)	
Parents’ attitude towards learning medical				
Support	1410 (69.1)	1270 (70.7)	95 (64.6)	0.197
Neutral	557 (27.3)	466 (25.9)	44 (29.9)	
Against	73 (3.6)	60 (3.3)	8 (5.4)	
Level of impact brought by COVID-19				
mean (SD)	3.5 (1.6)	3.5 (1.6)	3.8 (1.8)	**0.006**

*Abbreviations*: *CYN,* Currency in circulation in China.

^a^including 97 missing values

### Univariate analysis

After locking out missing values (*n* = 97), most of the participants (1796, 92.4%) maintained their career choice (i.e., 7.6% changed their choice). The chi-square test revealed that grade (*P* = 0.008) and regarding healthcare work as dangerous (*P* = 0.019) were correlated with changing their career choice during the COVID-19 pandemic. Grade 2 (61, 41.5%) and Grade 3 (30, 20.4%) had the largest number of UM students who changed their career choice, and 66% viewed healthcare work as dangerous. The degree of COVID-19’s impact presented a significant association (*P* = 0.006) with career choice. Those who indicated they may change their career choice had higher mean scores in the category of degree of impact, as compared with those who maintained their career choice. Additional details on univariate characteristics are presented in Table 1.

### Multiple logistic regression analysis

The variables that were observed to be significantly associated with a change of career choice during the COVID-19 pandemic were Grade 2 (*P* = 0.023), Grade 5 (*P* = 0.048), attitude towards medical career (*P* = 0.039), and having relatives with a medical background (*P* = 0.047). Compared with those in Grade 1, those who were in Grade 2 (adjusted odds ratio [aOR] 1.89, [95%CI, 1.09-3.26]) and grade 5 (aOR, 2.43 [95%CI, 1.01-5.86]) were more likely to change their career choice. Those who viewed healthcare as dangerous were more likely to change their career choice for the aOR 1.49 (95%CI [1.02-2.32]). Those who had relatives with a medical background were more likely to change the career choice (aOR,1.53 [95%CI, 1.01-2.32]). Additional details on logistic regression characteristics on career choice are shown in Table 2.

**Table 2:** Odds ratios (95%CI) of change of career intention for socio-demographic characteristics in logistic regression models

Variable	Adjusted OR (95%CI)	*P* (Wald’s test)	*P* (LR-test)
Age (year)	1.07 (0.91,1.27)	0.397	0.405
Sex			
Male	Reference		0.137
Female	0.72 (0.47,1.11)	0.133	
Residency			
Hubei rural	Reference		0.427
Hubei urban	0.82 (0.52,1.31)	0.414	
Rural other than Hubei	1.04 (0.63,1.72)	0.877	
Urban other than Hubei	0.6 (0.3,1.17)	0.135	
Educational level of parents			
Middle school or below	Reference		0.78
High school	1.12 (0.72,1.73)	0.616	
College	0.99 (0.49,2.00)	0.983	
University or above	1.32 (0.75,2.35)	0.337	
Family monthly income (CYN)			
<¥3000	Reference		0.55
¥3000-¥5999	1.02 (0.63,1.64)	0.937	
¥6000-¥11,999	0.79 (0.45,1.39)	0.416	
≥¥12,000	0.64 (0.28,1.47)	0.294	
Monthly expense (CYN)			
<¥500	Reference		0.53
¥500-¥999	0.59 (0.26,1.36)	0.217	
¥1000-¥1499	0.68 (0.3,1.58)	0.374	
≥¥1500	0.83 (0.32,2.15)	0.703	
Grade			
Grade 1	Reference		**0.014**
Grade 2	1.89 (1.09,3.26)	**0.023**	
Grade 3	1.15 (0.6,2.22)	0.673	
Grade 4	1.07 (0.5,2.29)	0.864	
Grade 5	2.43 (1.01,5.86)	**0.048**	
Major			
Clinical medicine	Reference		0.095
Nursing	1.29 (0.83,2.02)	0.256	
Others	0.62 (0.31,1.26)	0.186	
Time of concern about COVID-19 (minutes)			
<5	Reference		0.076
5-9	0.5 (0.29,0.87)	0.014	
10-29	0.71 (0.44,1.15)	0.166	
≥30	0.83 (0.49,1.41)	0.500	
Regard healthcare as a dangerous job			
No	Reference		**0.036**
Yes	1.49 (1.02,2.16)	**0.039**	
Have relatives with medical background			
No	Reference		**0.051**
Yes	1.53 (1.01,2.32)	**0.047**	
Parents’ attitude towards learning medical			
Support	Reference		0.232
Neutral	1.18 (0.8,1.75)	0.402	
Against	1.99 (0.9,4.41)	0.089	
Level of impact brought by COVID-19	1.11 (0.99,1.23)	0.065	0.067

*Abbreviations*: *CYN,* Currency in circulation in China

The degree of COVID-19’s impact was a common factor significantly associated with career preference (aOR, 1.17 [95%CI, 1.06-1.30]), career perspective (aOR, 1.08 [95%CI, 1.01-1.15]), and ideal workplace (aOR, 1.11 [95%CI, 1.02-1.21]). Factors associated with a change of career preference were Grade 2 (aOR, 2.21; [95%CI, 1.29-3.78]), personal expenses being between ¥500 to ¥999 per month (aOR, 0.39; [95%CI, 0.18-0.84]) and parents’ attitude towards their child being a UM student (aOR, 1.64 [95%CI, 1.13-2.38]). Compared with those with monthly expenses of less than ¥500, those whose expenses were ¥500 to ¥999 were less likely to change their career preferences (aOR, 0.39 [95%CI, 0.18-0.84]); however, no significant difference was observed amongst those whose expenses were higher or lower. Compared with those who had their parents’ support in attending medical schools, those who had parents who neither supported nor were against were more likely to change career preference (aOR, 1.64 [95%CI, 1.13-2.38]). Participants’ hometown (*P* = 0.018) and whether they had relatives with a medical background (*P* = 0.021) were associated with a change in career perspective. Compared with those who were from rural areas in Hubei, those whose hometowns were in urban areas were less likely to change their career perspective - whether they were from Hubei (aOR, 0.69 [95%CI, 0.52-0.92]) or not (aOR, 0.60 [95%CI, 0.41-0.89]). Participants who had relatives with a medical background (aOR, 1.37 [95%CI, 1.05-1.78]) were more likely to change their career preference. Age (*P* = 0.018) and major (*P* <  0.001) were associated with changing ideas about the ideal workplace. Higher ages were associated with a greater possibility of changing ideas about the ideal workplace (aOR, 1.17 [95%CI, 1.03-1.34]). Nursing UM students were more likely to change their idea of the ideal workplace than clinical UM students (aOR, 2.07 [95%CI, 1.42-3.03]). Additional details on logistic regression characteristics on career preference, career perspective, and ideal workplace are shown in Table 3.

**Table 3:** Multiple logistic regression analysis of factors associated with career preference,career perspective and workplace preference

Variable	Career preference		Career perspective		Ideal workplace	
	Adjusted OR(95%CI)	*P*	Adjusted OR (95%CI)	*P*	Adjusted OR(95%CI)	*P*
Age (year)	0.97 (0.82,1.16)	0.768	0.97 (0.87,1.08)	0.565	1.17 (1.03,1.34)	**0.018**
Sex						
Male	Reference	0.990	Reference	0.252	Reference	0.587
Female	1.00 (0.63,1.59)		1.17 (0.89,1.54)		0.90 (0.62,1.31)	
Residency						
Hubei rural	Reference	0.199	Reference	**0.018**	Reference	0.657
Hubei urban	1.02 (0.65,1.6)		0.69 (0.52,0.92)		0.83 (0.57,1.21)	
Rural other than Hubei	1.10 (0.66,1.84)		0.76 (0.56,1.03)		1.01 (0.68,1.52)	
Urban other than Hubei	0.51 (0.24,1.08)		0.60(0.41,0.89)		0.76 (0.45,1.30)	
Educational level of parents						
Middle school or below	Reference	0.344	Reference	0.879	Reference	0.136
High school	1.13 (0.73,1.76)		1.04 (0.80,1.35)		1.04 (0.73,1.47)	
College	1.35 (0.69,2.65)		1.07 (0.71,1.61)		0.74 (0.40,1.38)	
University or above	1.68 (0.96,2.92)		0.91 (0.64,1.31)		1.49 (0.96,2.33)	
Family monthly income (CYN)						
< ¥3000	Reference	0.187	Reference	0.448	Reference	0.329
¥3000-¥5999	0.71 (0.45,1.14)		0.88 (0.66,1.18)		0.94 (0.64,1.38)	
¥6000-¥11,999	0.53 (0.31,0.94)		0.81 (0.58,1.13)		0.76 (0.48,1.19)	
≥¥12,000	0.60 (0.26,1.36)		1.06 (0.65,1.72)		0.58 (0.29,1.15)	
Monthly expense (CYN)						
<¥500	Reference		Reference		Reference	
¥500-¥999	0.39 (0.18,0.84)	**0.015**	1.66 (0.86,3.21)	0.282	0.52 (0.26,1.01)	0.076
¥1000-¥1499	0.67 (0.31,1.46)		1.41 (0.72,2.75)		0.61 (0.31,1.21)	
≥¥1500	0.44 (0.17,1.14)		1.36 (0.66,2.82)		0.88 (0.41,1.90)	
Grade						
Grade 1	Reference	**0.024**	Reference	0.605	Reference	0.200
Grade 2	2.21 (1.29,3.78)		1.16 (0.86,1.58)		1.54 (1.00,2.37)	
Grade 3	1.30 (0.67,2.55)		1.09 (0.75,1.57)		1.30 (0.79,2.13)	
Grade 4	1.64 (0.77,3.50)		1.25 (0.81,1.93)		1.04 (0.58,1.85)	
Grade 5	1.87 (0.69,5.02)		1.51 (0.86,2.67)		1.13 (0.51,2.47)	
Major						
Clinical medicine	Reference	**0.021**	Reference	0.108	Reference	**< 0.001**
Nursing	1.41 (0.90,2.21)		1.32 (1.01,1.73)		2.07 (1.42,3.03)	
Others	0.53 (0.24,1.16)		1.05 (0.73,1.51)		1.04 (0.61,1.77)	
Time of concern about COVID-19(minutes)						
<5	Reference	0.216	Reference	0.945	Reference	0.919
5-9	0.64 (0.38,1.08)		1.00 (0.72,1.40)		0.85 (0.55,1.32)	
10-29	0.59 (0.36,0.97)		1.05 (0.77,1.43)		0.91 (0.60,1.37)	
≥30	0.72 (0.42,1.23)		0.96 (0.67,1.36)		0.92 (0.58,1.46)	
Have relatives with medical background						
No	Reference	0.390	Reference	**0.021**	Reference	0.811
Yes	1.21 (0.78,1.88)		1.37 (1.05,1.78)		1.05 (0.73,1.50)	
Parents’ attitude toward learning medical						
Support	Reference	**0.031**	Reference	0.588	Reference	0.200
Neutral	1.64 (1.13,2.38)		1.06 (0.84,1.35)		1.32 (0.97,1.79)	
Against	0.82 (0.28,2.37)		0.78 (0.43,1.42)		0.99 (0.46,2.14)	
Level of impact	1.17 (1.06,1.30)	**0.003**	1.08 (1.01,1.15)	**0.016**	1.11 (1.02,1.21)	**0.018**

*Abbreviations*: *CYN,* Currency in circulation in China

### Stratified analysis

**Fig. 1:**
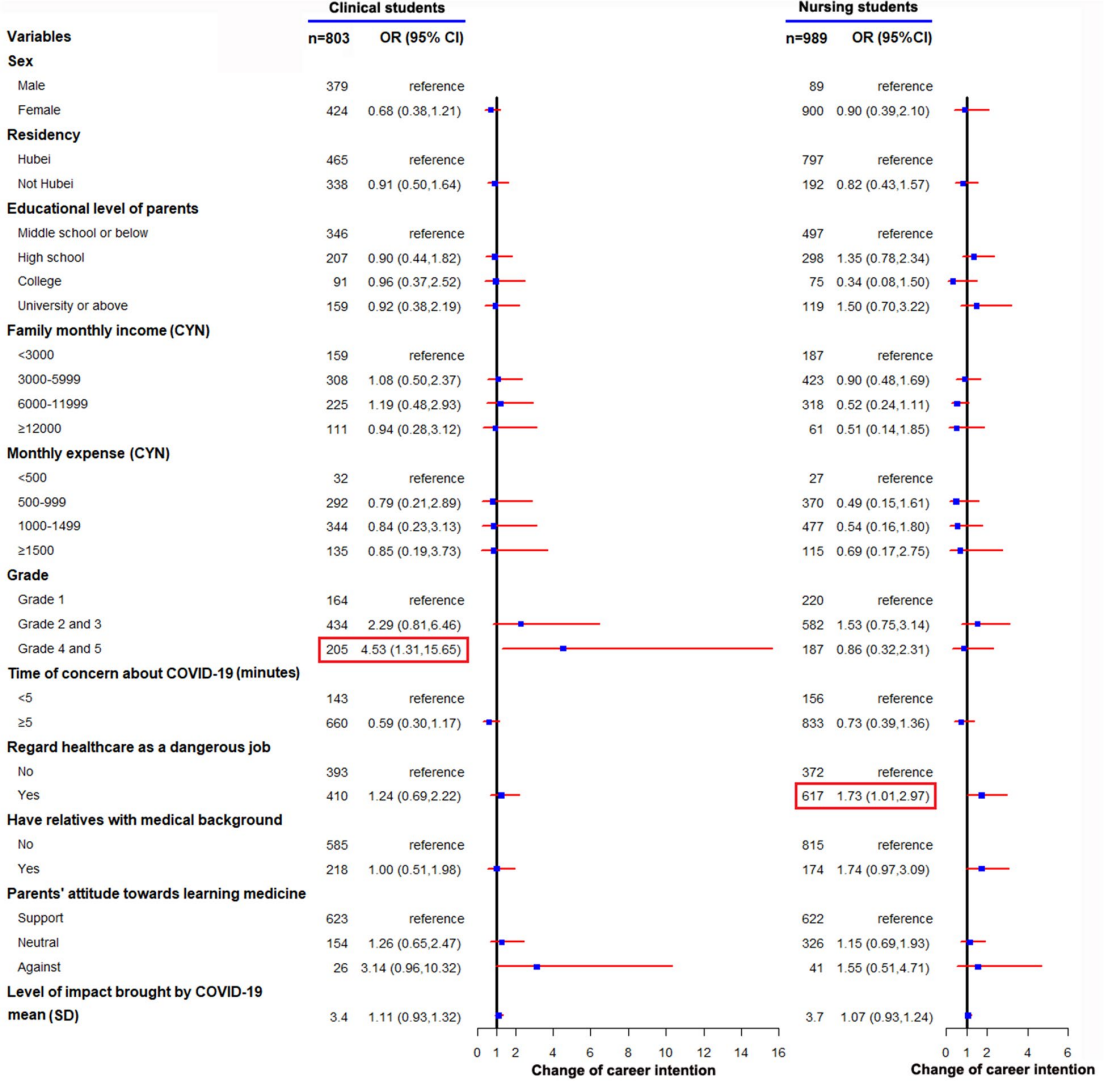
Stratified analysis of factors associated with career choice by UM students’ major

We performed a stratified analysis by majors based on changes in career choice. Multiple logistic regression analyses identified that the clinical UM students in Grades 4 and 5 were more likely to change the career choice (aOR 4.53 [95%CI 1.31-15.65]; Fig. 1), and seeing healthcare work as dangerous was associated with nursing UM students’ changing their career choice (aOR 1.73 [95%CI 1.011-2.97]; Fig. 1). The detailed results of the stratified analysis are shown in Fig. 1.

## Discussion

Our results are consistent with our hypothesis that the COVID-19 pandemic influenced UM students’ career intentions. As we know, health workers are always under heavy pressure and have a high workload. Undergraduate medical students have witnessed at first hand the fight against the pandemic, which caused much more pressure and an even heavier workload, particularly considering the lack of mature healthcare workers. They eventually realized the urgent need for a medical frontline even when they knew they would face a heavy burden later on. Previous studies have shown that ordinary people, including healthcare workers, suffered from this large-scale infectious public health event and experienced unprecedented pressure and enormous disruptions to their daily lives [14, 15]. Moreover, the pandemic reflected not only the high risk of occupational exposure but also the urgent need for trained health workers [16].

However, role models gave the frontline a sense of professional belonging. A Japanese research study found that although the pandemic influenced the overall performance of the medical professions, all participants showed a sense of belonging [17]. Faced with challenges and opportunities, it was highly likely that some UM students would change their career intentions.

The present study found that the participants from Grades 2 and 5 were more likely to change their career choice than those in Grade 1. Grade was an important factor in changes in career preference. Similarly, Indian medical students changed their career preferences and motivational factors in the later stages of their degree programmes [18]. Studies have shown that public health emergencies negatively impact students’ mental health [19]. Grade 5 students need to balance the assignment of an internship and postgraduate entrance exam preparation. If they fail the examination, they must either join the Resident Physician Standardized Training in China for 3 years or find another job. Moreover, social distance changed the nature of the students’ interactions with mentors [20], and they had little counselling or mental support. Hence, the Grade 5 students were more likely to change their intention of becoming healthcare workers. By contrast, Grade 2 students studied elementary courses at home during the pandemic. They were protected from clinical diagnosis and treatment work because they were isolated. Thus, potential sources of inspiration and guidance were vital for them if they were to maintain their enthusiasm and confidence in learning. There should be different guidelines for different grades. For example, Grade 1 students should build up an interest in medical knowledge. Grade 2 students, before embarking on internships, should enhance their basic knowledge and theoretical understanding. Trainees should acquire more basic skills by participating in clinical daily work, keeping in contact with their clinical instructors, and building an appropriate career plan and development ideology.

In the descriptive analyses, the mean score for the level of impact of the pandemic on the participants was moderate. The adverse effects were a driving factor for changes in career choice, career preference, career perspectives, and workplace preference. Other researchers have made similar observations [20], suggesting that the pandemic changed trainees’ daily activities due to social isolation. With the remission of the pandemic, routine life is back on track. However, the psychological damage that has been caused will take time to be addressed. In addition, “regard healthcare as a dangerous job” and “have relatives with medical background” were the influence factors for changing career intention. Other studies have shown that medical students perceived fear [21] and negative feedback from families and friends concerning frontline work [22], and these were risk factors for changes in career intention. Thus, psychological counselling is vital for UM students.

In the present study, changes in career preference were associated with monthly expenses, grade, the type of major, parental support, and the level of impact of the pandemic. Ruban et al. reported that the career preference was associated with gender, whether participants had family members in the health professions, and residency background [18]. We suspected that the difference was because the pandemic outbreak threatened the whole population. As was noted, we found that the type of major was a driving factor; for example, nursing students were more inclined to want to change their ideal working place compared with clinical students, possibly because they found it easier to find jobs. A study of nursing students’ career choices following the COVID-19 demonstrated that the pandemic appeared to have had a positive effect on the choice to study nursing because of positive media reports of the profession [23]. These, along with social support, may give health professionals a sense of belonging.

With the virus variation, and the reappearance of sporadic cases in China, it is hard to forecast when the pandemic will be controlled. Biomedical experts have claimed that the virus keeps changing and pandemics will be part of what has been termed the new normal [24]. One research study found that Hurrican Katrina disrupted medical education and led to significant changes in specialty choices both before and after the event, and this change was evident over a 10-year study period [25]. Because it was a cross-sectional retrospective study, the causal relationships could not be determined. Thus, it is difficult to tell at this stage whether the career-related concerns revealed by our findings will manifest as actual changes in career intentions.

Few studies have investigated the pandemic’s impact on medical students’ career intentions, though it seems that it has affected medical students’ career perceptions [26]. Medical education systems vary according to country, so further studies are needed that explore the association of the pandemic with medical students’ career intentions and its long-term influences. Personal career intentions are jointly affected by intrinsic value factors (such as interests and professional identity) and external value factors (such as career stability, income, and welfare) [13]. Vocational risk education and the prevention of occupational exposure should be standard for medical students and frontline health workers. It is also vital to strengthen public health education; with people’s recognition of the profession, medical students are more likely to become health workers [27, 28]. Administrators should increase investment in public health and enhance primary protection through public health information campaigns.

### Strengths and limitations of the present study

For this study, we recruited a large sample of participants by describing the survey to potential respondents in neutral terms and making it short and easy to complete. Although the sample was self-selected, the participants came from different grades, familial economic circumstances, birthplaces, and levels of parental education. We used rigorous post-stratification weighting that enabled us to extrapolate students’ career intentions from different specialties.

The study had several limitations. First, its cross-sectional nature meant that causality cannot be proven. Further research is needed to clarify any associations. Second, we used only one centre, so this may have resulted in selection bias, and the results cannot be generalized to the Chinese population. Finally, because the data were collected through an online questionnaire via WeChat, the respondents made a personal decision to participate, which raises questions regarding self-selection.

## Conclusion

Changes in career intention were particularly influenced by grade, attitude towards being a health worker, and COVID-19’s impact on the participants’ lives. Educators should give students more detailed employment guidance, which would help to reduce medical brain drain and produce high-quality staff.

## Supplementary Information

**Additional file 1: Supplementary Table 1.** The score of effect of the COVID-19 pandemic on students’ life.

**Additional file 2: Supplementary Table 2.** The correlations between each item.

## Data Availability

The datasets of this article are available from the corresponding author on a reasonable request.
