# Supplementary material for: Impact of the COVID-19 pandemic on career intention amongst undergraduate medical students: a single-centre cross-sectional study conducted in Hubei Province
Source: BMC Med Educ. 2022 Mar 8;22:154. doi: 10.1186/s12909-022-03201-4 (PMC8901388; doi:10.1186/s12909-022-03201-4)
Supplement: Supplementary file 1 — Additional file 1: Supplementary Table 1. The score of effect of the COVID-19 pandemic on students’ life. [file 12909_2022_3201_MOESM1_ESM.docx]

Supplementary table1 The score of effect of the COVID-19 pandemic on students’ life.

| Variables | Yes | No |
| --- | --- | --- |
| learning environment | 1 | 0 |
| sleep quality | 1 | 0 |
| diet | 1 | 0 |
| travel ability | 1 | 0 |
| happiness | 1 | 0 |
| life routine | 1 | 0 |
| psychology | 1 | 0 |
| Sum score | 7 | 0 |
