# Supplementary material for: Impact of the COVID-19 pandemic on career intention amongst undergraduate medical students: a single-centre cross-sectional study conducted in Hubei Province
Source: BMC Med Educ. 2022 Mar 8;22:154. doi: 10.1186/s12909-022-03201-4 (PMC8901388; doi:10.1186/s12909-022-03201-4)
Supplement: Supplementary file 2 — Additional file 2: Supplementary Table 2. The correlations between each item. [file 12909_2022_3201_MOESM2_ESM.docx]

Supplementary Table 2 The correlations between each item.

|  | Impact 1 | Impact 2 | Impact 3 | Impact 4 | Impact 5 | Impact 6 | Impact 7 |
| --- | --- | --- | --- | --- | --- | --- | --- |
| Impact 1 | 1.00 | 0.26 | 0.16 | 0.15 | 0.00 | 0.07 | 0.07 |
| Impact 2 | 0.26 | 1.00 | 0.32 | 0.28 | 0.00 | 0.16 | 0.24 |
| Impact 3 | 0.16 | 0.32 | 1.00 | 0.25 | -0.04 | 0.15 | 0.23 |
| Impact 4 | 0.15 | 0.28 | 0.25 | 1.00 | 0.20 | 0.22 | 0.11 |
| Impact 5 | 0.00 | 0.00 | -0.04 | 0.20 | 1.00 | 0.18 | 0.00 |
| Impact 6 | 0.07 | 0.16 | 0.15 | 0.22 | 0.18 | 1.00 | 0.13 |
| Impact 7 | 0.07 | 0.24 | 0.23 | 0.11 | 0.00 | 0.13 | 1.00 |
